# Inhibitory effects of a hot-water extract of cumin fruit on influenza A virus infection

**DOI:** 10.1371/journal.pone.0326423

**Published:** 2025-06-27

**Authors:** Abdullah Al Sufian Shuvo, Yoshihiko Maekawa, Masahiro Kassai, Takeshi Kawahara

**Affiliations:** 1 Division of Food Science and Biotechnology, Department of Science and Technology Agriculture, Graduate School of Medicine, Science and Technology, Shinshu University, Nagano, Japan; 2 Department of Animal Nutrition, Bangladesh Agricultural University, Mymensingh, Bangladesh; 3 Department of Agriculture, Graduate School of Science and Technology, Shinshu University, Nagano, Japan; 4 Central Research Institute, S&B Foods Inc., #605 Mitsui Link-Lab Shinkiba1 Shinkiba 2-3-8, Koto-ku, Tokyo, Japan; 5 Academic Assembly, School of Science and Technology, Institute of Agriculture, Shinshu University, Nagano, Japan; Taif University, SAUDI ARABIA

## Abstract

The influenza A virus (IAV) is an extremely contagious virus responsible for both seasonal flu and global pandemics. Cumin (*Cuminum cyminum* L., family Apiaceae) is a spice widely used in numerous Asian nations. The cumin fruit, commonly termed ‘cumin seed’, has been used in traditional medicine for the treatment of several ailments; however, its effect on IAV is not completely understood. This study investigated the effect of cumin fruit hot-water extract (CWE) on IAV infection. Madin-Darby canine kidney (MDCK) cells were infected with IAV (H1N1) and used for *in vitro* experiments. Pre-infection treatment of the target cells with CWE suppressed M1 protein expression in a dose-dependent manner, whereas post-infection treatment had no such effect. CWE at concentrations of 12.5 µg/mL or higher also inhibited IAV-induced haemagglutination and clathrin-dependent endocytosis. Even, a plaque formation assay was also conducted to confirm the efficacy of CWE on virus replication. The assay results showed that CWE significantly reduced IAV replication. However, the expression of type I interferon (IFN) and IFN-stimulated antiviral protein genes was not affected by CWE in the virus-infected cells. Furthermore, the presence of cuminaldehyde in CWE was examined using high-performance liquid chromatography. Cuminaldehyde was not detected in the CWE used in this study. Moreover, cells that were pre-treated with a cuminaldehyde standard did not show any inhibition of IAV infection. The current *in vitro* study showed that CWE inhibited IAV infection without harming host cells. Thus, CWE may be used to prevent IAV infections by limiting viral attachment and absorption.

## Introduction

Influenza viruses are negative-strand RNA viruses belonging to the *Orthomyxoviridae* family and are primarily responsible for highly contagious episodes of seasonal flu. These viruses are classified into three types (A, B, and C) based on differences in their nucleoprotein and matrix protein; type A and B viruses can infect humans and cause seasonal epidemics [1]. The influenza A virus (IAV) can be characterised by the pattern of the antigenicity of haemagglutinin (HA) and neuraminidase (N) proteins of the viral membrane [2]. IAV is known to cause global pandemics when new subtypes appear due to mutations. Millions of people have died over the course of four influenza pandemics in the last century: H1N1 (1918), H2N2 (1957), H3N2 (1968), and H1N1 (2009) [3]. Moreover, influenza pandemics create major health concerns owing to their high morbidity and mortality rates. Influenza can affect people of all ages, and annually, 10% of people worldwide are affected by influenza [4]. Different types of medications are used to prevent influenza infection worldwide; however, variations produced from genetic drift and shift and the emergence of new variants resistant to existing antiviral therapies pose significant challenges. Generally, M2 ion channel inhibitors like amantadine, rimantadine and neuraminidase inhibitors like oseltamivir, zanamivir are used to treat influenza infections, but the influenza virus is becoming resistant to these drugs, which is big problem right now. To provide antiviral protection, many researchers have focused on the functionality of food products and investigated the mechanism of action of food components with antiviral activity. Certain dietary chemical components have been shown to provide protection against viral diseases by limiting viral infection and enhancing antiviral immune functions. As described by Viuda-Martos *et al.*, spices, which have long been employed in traditional medicine in many countries [5], may have potential as antiviral materials, and research in the pharmaceutical, chemical, and food industries may facilitate better utilisation of the physiological effects of spices. In addition, the secretion of IFN-β, a key factor in stimulating numerous interferon-stimulated proteins that suppress influenza infection, can be enhanced by bioactive components found in different plants and spices [6,7].

Cumin (*Cuminum cyminum* L.), a member of the Apiaceae family, is a tiny, aromatic, annual spice and is the second-most popular spice globally after black pepper [8]. Cumin is grown in India, the Middle East, China, and Mediterranean countries [9,10]. The distinctive flavour and aroma of this spice makes it a versatile ingredient in many recipes. Moreover, the fruit of cumin, commonly termed ‘cumin seed’, has long been used in traditional medicine and is prescribed for a wide range of human ailments [11,12]. Some conditions that can be treated with cumin fruits include corneal opacities, ulcers, toothache, diarrhoea, epilepsy, jaundice, inflammation, and fever [11,13–15]. Cumin fruits have been shown to possess antibacterial, anti-inflammatory, anti-allergic, immunomodulatory, anti-amyloidogenic, and antifungal properties [16,17]. However, little is known about the effects of cumin on influenza virus infection.

In this study, we evaluated the anti-influenza activity of a cumin fruit hot-water extract (CWE) in an *in vitro* infection model using an IAV strain. We evaluated the effect of pre-infection treatment of target cells with CWE on the replication of viral protein. We further investigated the effect of CWE on the IAV adsorption and invasion process.

## Materials and methods

### Preparation of CWE

The fruits of *C. cyminum* were harvested in Turkey. Detailed information of the plant material with collection data has been deposited in S&B Foods Inc. (Tokyo, Japan, Lot No. 20170725). To obtain CWE, 100 mL of water was added to 10 g of cumin fruits, and extraction was performed at 100°C for 1 h while stirring. The resulting extract was centrifugally filtered through 5A filter paper (Advantec, Tokyo, Japan), and the supernatant was lyophilised for use as CWE. CWE was dissolved in water or 50% ethanol and used for subsequent experiments.

### Cells and viruses

Madin-Darby canine kidney (MDCK) cells were obtained from the Japanese Collection of Research Bioresources (JCRB) Cell Bank (Osaka, Japan), maintained in Eagle’s Minimal Essential Medium (EMEM; FUJIFILM Wako Pure Chemical, Osaka, Japan) supplemented with heat-inactivated 10% (v/v) foetal bovine serum (FBS; Cytiva, Tokyo, Japan) and 100 U/mL penicillin-100 μg/mL streptomycin solution (FUJIFILM Wako Pure Chemical), and cultured in a humidified atmosphere of 5% CO_2_ at 37°C. The human IAV strain A/Puerto Rico/8/34 (H1N1) was obtained from the American Type Culture Collection (Manassas, VA, USA) and propagated in MDCK cells. For infection experiments, the virus was activated by treating with 100 μg/mL acetylated trypsin (Merck, Darmstadt, Germany) in sterilised phosphate-buffered saline (PBS; pH 7.2) for 1 h at 37°C.

### Cytotoxicity assay

MDCK cells were seeded into a 96-well cell culture plate (Corning, Corning, NY, USA) at 4.0 × 10^4^ cells/100 μL/well and incubated for 24 h. After incubation, the medium was replaced with EMEM containing 10% FBS along with different concentrations of CWE (0–100 µg/mL) and incubated for another 24 h_._ After the treatment, 20 µL (5 mg/mL) 3-(4,5-dimethylthiazol-2-yl)-2,5-diphenylterazolium bromide (MTT) solution (Dojindo, Kumamoto, Japan) was added and incubated for another 4 h. Formazan was then solubilised by adding 100 µL dimethyl sulfoxide (FUJIFILM Wako Pure Chemical). The absorbance at 490 nm was measured using an iMark microplate reader (Bio-Rad, Hercules, CA, USA) to quantify the viable cells.

### Viral infection inhibition assay

**Fig 1:**
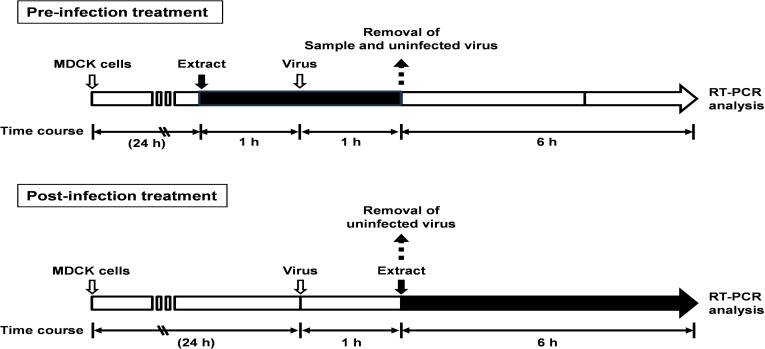
Protocol of the time-of-addition assay. Pre-infection treatment: before virus infection, Madin-Darby canine kidney (MDCK) cells were treated with CWE at different concentrations for 1 h and infected with virus for another 1 h. After removing the CWE and viruses, cells were cultured for 6 h. Post-infection treatment: MDCK cells were infected with viruses for 1 h. After removing the virus, cells were cultured in the presence of CWE for 6 h.

The effect of CWE on viral infection was evaluated using a time-of-addition assay as shown in Fig 1. MDCK cells were seeded into 24-well cell culture plates (Thermo Fisher Scientific, Waltham, MA, USA) at a density of 2 × 10^5^ cells/mL/well. After 24 h, 2 µL CWE (0–100 µg/mL) was added to 200 µL FBS-free EMEM solution and incubated for 1 h. After incubation, 2 µL diluted activated viral suspension (2 × 10^3^ PFU/mL) was used for infection. After 1 h of infection, the viral inoculum was removed, and the cells were washed with sterilised PBS. After culturing the cells in 10% FBS-supplemented EMEM for another 6 h, they were lysed using TRI reagent (Merck).

### Quantitative reverse transcription PCR (RT-PCR)

Total RNA was isolated from cell lysates in accordance with the manufacturer’s instructions and reverse-transcribed at 42°C for 50 min using the M-MLV reverse transcriptase kit (Thermo Fisher Scientific) and 10 pmol/μL random primer pd (N)_9_ (Takara Bio, Shiga, Japan) in a thermal cycler (PTC-200; MJ Research, Waltham, MA, USA). The resultant cDNA was then subjected to real-time RT-PCR analysis with TB Green Premix Ex Taq II (Takara Bio) using the Eco Illumina Real-Time PCR system (Illumina, San Diego, CA, USA). The reaction consisted of one cycle of preheating (95°C, 10 s; 95°C, 10 min), 40 cycles of denaturation (95°C, 10 s), and primer annealing/extension (55°C, 30 s). The nucleotide sequences of the forward and reverse primers are listed in Table 1. Results were analysed with the ΔΔCt method using the Eco system software (Illumina). The amount of PCR product was normalised to GAPDH expression level.

**Table 1:** Primer sequences used in this study.

Target protein	Sequence (5′-3′)		Size (bp)
Influenza virus M1	Forward	CATGGAATGGCTAAAGACAAGAC	189
	Reverse	GCGTGAACACAAATCCTAAAATC	
Canine GAPDH	Forward	TTCCACGGCACAGTCA	115
	Reverse	ACTCAGCACCAGCATC	
Canine IFN beta	Forward	ATCCCTGAGGAAATCGAGAAA	161
	Reverse	CAGTGGAGCTTCACAAGAAGG	
Canine Mx1	Forward	TGTTGATATGCTGCACACGA	160
	Reverse	TCGGATGGACTTCTCAGCTT	
Canine OAS1	Forward	CACAGACCCAACCCTGAAGT	164
	Reverse	TGGTACCAGTGCTTGACGAG	
Canine ISG15	Forward	TCTGTGCCCCTGGAGGACTTGA	131
	Reverse	TGCTGCTTCAGCTCTGATGCCA	
Canine PKR	Forward	TGCTTTGGGGCTAATTCTTG	89
	Reverse	TGCCAGCCCTTAGCTCTTTA	

M1: Matrix protein 1; GAPDH: Glyceraldehyde-3-phosphate dehydrogenase; Mx1: Myxoma resistance; OAS1: 2′-5′-Oligoadenylate synthetase 1; ISG15: IFN-stimulated gene 15; PKR: Protein kinase R.

### Haemagglutination inhibition assay

Chicken erythrocytes (Kohjin Bio, Saitama, Japan) were washed thrice with sterilised PBS and suspended at 2% (v/v) in PBS. The erythrocyte suspension (25 µL) was added to a 96-well U-shaped microplate (Thermo Fisher Scientific). Then equal volumes (12.5 µL) of different concentrations (0–100 µg/mL) of CWE and activated IAV were added to each well. The mixture was then incubated at room temperature for 30 min to observe erythrocyte aggregation on the plate. The haemagglutination inhibition titre was calculated as the reciprocal of the highest dilution that produced complete haemagglutination inhibition.

### Endocytosis inhibition assay

MDCK cells were seeded in a 24-well plate (Thermo Fisher Scientific) at 1.0 × 10^5^ cells/mL/well and allowed to grow for 24 h. The cells were then washed with 1 mL live imaging solution (Thermo Fisher Scientific) three times and incubated for 1 h in 200 µL live cell imaging solution containing different concentrations (0–100 µg/mL) of CWE dissolved in water or 50% ethanol. Next, pHrodo Red Transferrin conjugate (Thermo Fisher Scientific) was added at a concentration of 10 µg/mL, followed by incubation for 15 min in a 5% CO_2_ incubator. After removing the solution and washing the cells with sterile PBS, cells were detached using 200 µL TrypLE Express (Thermo Fisher Scientific). The detached cells were washed by centrifugation at 10,000 rpm for 5 min and resuspended in sterile ice-cold PBS. A total of 10,000 cells were analysed using a FACSCelesta (BD Life Sciences, San Jose, CA, USA) at 560-nm excitation light irradiation. All data were analyzed using FACSDiva software (BD Life Sciences).

### Plaque formation assay

The impact of CWE on plaque formation by IAV was investigated by seeding MDCK cells (5.0 × 10^5^ cells/well) in a 6-well plate and allowing them to form a cell monolayer for 24 h in a humidified atmosphere of 5% CO_2_ at 37°C. The cell monolayer was washed with PBS. Following this, 500 µL of FBS-free EMEM containing varying concentrations of CWE (0–100 µg/mL) was introduced into the well and incubated for 1 h. After the pretreatment of the cell monolayer with CWE, 500 µL of serially diluted virus was then added. Following incubating for an additional 1 h, the cell monolayer was washed twice with FBS-free EMEM. Subsequently, the overlay media was added, which contained equal volume of 2 × MEM (GIBCO Life technologies, USA), 2% Bacto Agar (BD, 214010, USA), 0.02% bovine albumin serum (Sigma-Aldrich, St. Louis, MO, USA), 0.025% HEPES (GIBCO Life technologies), and 1 µg/mL tosyl-phenylalanine chloromethyl ketone (TPCK)-treated trypsin (Merck) and allowed to incubate at 37^o^C for 72 h. The cells were fixed with 10% formaldehyde (Nacalai tesque, Kyoto, Japan) for 1 h and stained with 1% crystal violet (Sigma-Aldrich) to count the number of plaques formed.

### High-performance liquid chromatography (HPLC)

HPLC analysis of CWE was performed using the 1260 infinityⅡPrime LC system (Agilent Technologies, Santa Clara, CA, USA) equipped with a InertSustain C18 column (3 μm, 4.6 mm × 100 mm; GL Sciences, Tokyo, Japan), 1260 Infinity II Flexible Pump, and 1260 Infinity II Multicolumn Thermostat (Agilent Technologies). The column temperature was 40°C and flow rate was set to 1.0 mL/min. Elution was conducted by a gradient method using 0.1% formic acid in methanol/H_2_O as follows: 2/98 (v/v) (0 min), 5/95 (2–10 min), 40/60 (30 min), 98/2 (50–55 min), and 2/98 (55.1–60 min). Cuminaldehyde was detected at 265 nm by comparison with the retention time of a commercially available cuminaldehyde (Tokyo Chemical Industry, Tokyo, Japan) as a reference standard using a photodiode array detector (1260 Infinity II Diode Array Detector WR; Agilent Technologies).

### Statistical analysis

The data were statistically analysed using one-way analysis of variance (ANOVA) with Tukey-Kramer multiple-comparison tests in Excel 2019 (Microsoft, Redmond, WA, USA) with the add-in software Statcel4 (OMS Publishing, Tokyo, Japan). Results with a *p*-value of less than 0.05 were considered statistically significant.

## Results

### Yield of CWE

In this study, a hot water extraction process was used to obtain various biologically active ingredients different from oil extraction. CWE was obtained as a light-yellow powder. The yield (%) of CWE was 18.1% from cumin fruits on a dry basis. The obtained CWE was stored at 4°C until further use.

### Cytotoxicity of CWE against MDCK cells

**Fig 2:**
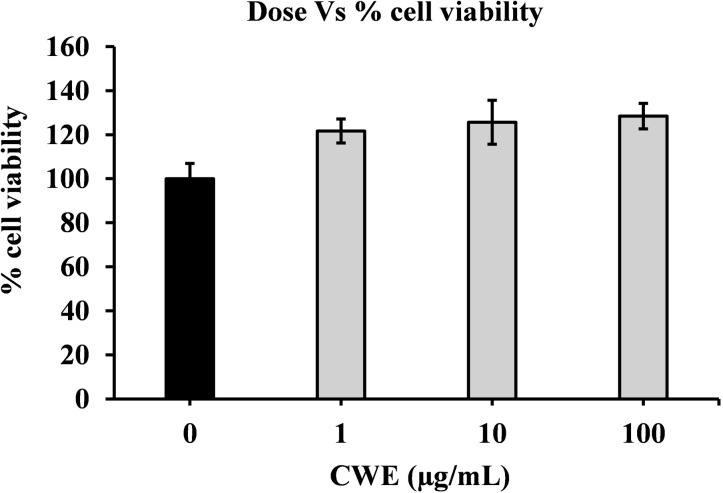
Cytotoxicity of CWE against MDCK cells. Cytotoxicity of CWE at concentrations of 1, 10, and 100 μg/mL against MDCK cells. Data are presented as relative mean ± standard deviation (SD) against CWE-untreated control (0 μg/mL) (n = 3).

The cytotoxicity of CWE against MDCK cells was evaluated using the MTT assay. The results indicated that CWE did not exhibit cytotoxic effects on the MDCK cells at concentrations ranging from 0 to 100 μg/mL (Fig 2), indicating that the antiviral properties indicated by this concentration were not due to cytotoxicity. Thus, CWE at concentrations within the range of 1 and 100 µg/mL was used for subsequent investigations.

### Effects of CWE on viral infections

To evaluate the effect of CWE on IAV replication, MDCK cells were exposed to CWE (dissolved in 50% ethanol or water) before and after viral infection. Subsequently, the cells were collected and analysed using RT-PCR to measure the expression level of the matrix protein 1 (M1).

**Fig 3:**
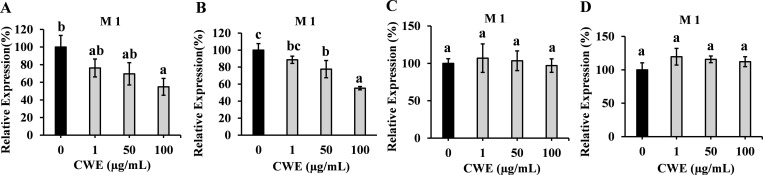
Effects of CWE on IAV infection. Viral M1 protein expression in cells that received pre-infection treatment with CWE dissolved in 50% ethanol (A) and water (B) or post-infection treatment with CWE dissolved in 50% ethanol (C) and water (D) at concentrations of 0, 1, 50, 100 μg/mL. Data are presented as relative mean ± SD against CWE-untreated control (0 μg/mL) (n = 3). Different letters denote a significant difference at *p* < 0.05.

M1 expression showed a significant dose-dependent reduction in cells that were treated with CWE before infection (Fig 3). These findings were not influenced by the solvents (50% ethanol or water) used to dissolve the CWE. In comparison with its expression in the untreated control (0 µg/mL), M1 protein expression reduced by approximately 24% when cells were pre-treated with 1 µg/mL CWE dissolved in 50% ethanol. Furthermore, CWE concentrations of 50 and 100 µg/mL resulted in reductions of 30% and 45%, respectively, of M1 expression in comparison with the untreated control (0 µg/mL). Moreover, cells pre-treated with CWE dissolved in water at concentrations of 1, 50, and 100 µg/mL reduced M1 protein expression by 11%, 22%, and 44%, respectively, in comparison with the untreated control (0 µg/mL). In contrast, post-infection treatment with CWE dissolved in 50% ethanol or water did not decrease M1 protein levels.

### Effects of CWE on haemagglutination

**Fig 4:**
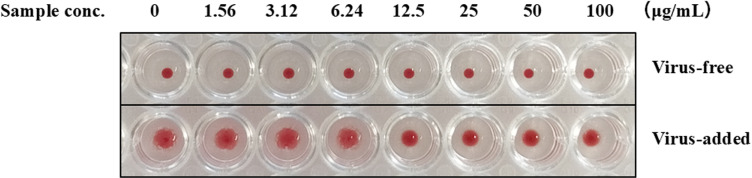
Effects of CWE on haemagglutination. Haemagglutination inhibitory activity of serially diluted concentrations of CWE against influenza A virus. Red blood cells (RBCs) were treated with CWE in the presence or absence of influenza A virus. In virus-free panel, RBCs were incubated with only CWE (0-100 µg/mL), whereas in the virus-added panel RBCs were incubated with CWE (0-100 µg/mL) along with influenza virus.

To investigate the effect of CWE on the initial phase of the viral life cycle, a haemagglutination inhibition assay was conducted to determine its ability to disrupt the haemagglutination of red blood cells (RBCs) caused by influenza virus infection. Considering the observed suppression of M1 protein by CWE, we performed a haemagglutination inhibition assay to assess the potential of CWE to impede haemagglutination, thereby restricting viral entry into cells. The results presented in Fig 4 indicated that haemagglutination was inhibited by CWE at concentrations ≥ 12.5 µg/mL.

### Effects of CWE on clathrin-dependent endocytosis

**Fig 5:**
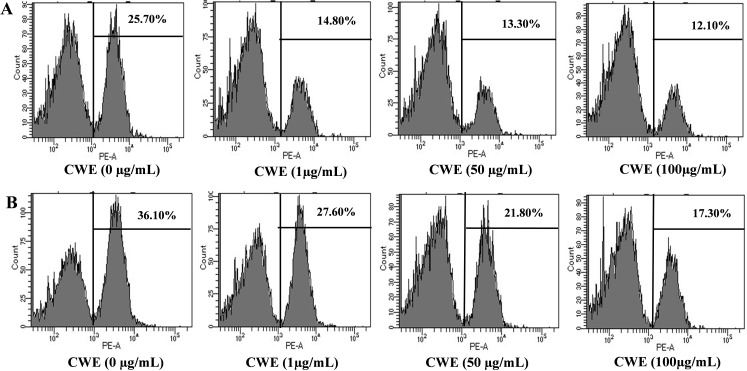
Effects of CWE on endocytosis. Cells were treated with CWE dissolved in 50% ethanol (A) or water (B). Data are shown as a single-parameter histogram representing 10,000 cells in flow cytometric analysis. Percentage designates the ratio of cell counts in positive area.

In this experiment, the pHrodo Red Transferrin conjugate was used to observe the inhibitory effects of CWE on clathrin-dependent endocytosis. CWE dissolved in 50% ethanol or water inhibited endocytosis in a dose-dependent manner (Fig 5). Cells pre-treated with 1, 50, and 100 µg/mL CWE dissolved in 50% ethanol showed 42.41%, 48.24%, and 52.91%, respectively, endocytosis inhibition compared with untreated control (0 µg/mL). In addition, cells pre-treated with 1, 50, and 100 µg/mL CWE diluted in water showed 23.54%, 39.61, and 52.07%, respectively, inhibition of endocytosis compared with untreated control (0 µg/mL).

### Effects of CWE on the expression of interferon (IFN)-β and IFN-stimulated proteins

To determine whether the suppression of the influenza virus was mediated by the induction of IFN-β and IFN-stimulated antiviral proteins by CWE, we measured the expression levels of these target proteins in cells 6 h after infection.

**Fig 6:**
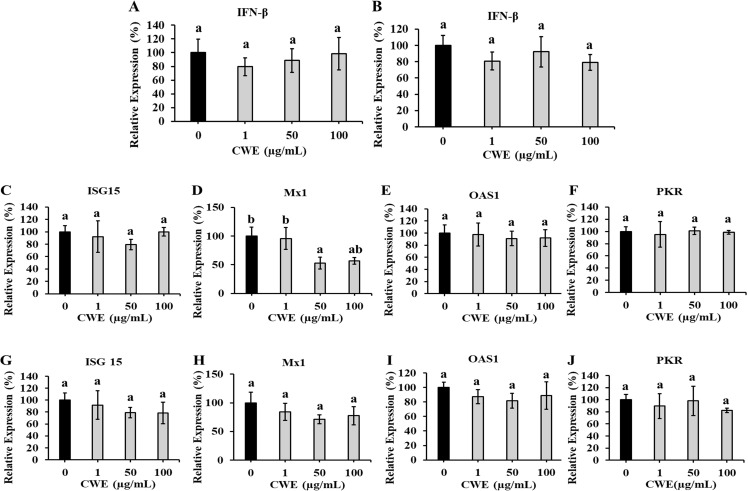
Effects of CWE on the expression of interferon (IFN)-β and IFN-stimulated antiviral proteins. The expressions of IFN-β in cells treated with CWE (pre-infection treatment) dissolved in 50% ethanol (A) or water (B) at concentrations of 0, 1, 50, 100 μg/mL and IFN-stimulated antiviral proteins in cells treated with CWE (pre-infection treatment) dissolved in 50% ethanol (C–F) or water (G–J) at concentrations of 0, 1, 50, 100 μg/mL. Data are presented as relative mean± SD against CWE-untreated control (0 μg/mL) (n = 3). Different letters denote a significant difference at *p* < 0.05.

CWE dissolved in 50% ethanol or water did not affect the production of IFN-β (Fig 6), which is reported to be decreased in MDCK cells after influenza virus infection [18]. CWE also had no significant effect on the expression of IFN-stimulated antiviral proteins, including IFN-stimulated gene 15 (ISG15), Myxoma resistance 1 (Mx1), 2′-5′-oligoadenylate synthetase 1 (OAS1), and protein kinase R (PKR), except for a slight downregulation of Mx1.

### Effect of CWE on virus replication

**Fig 7:**
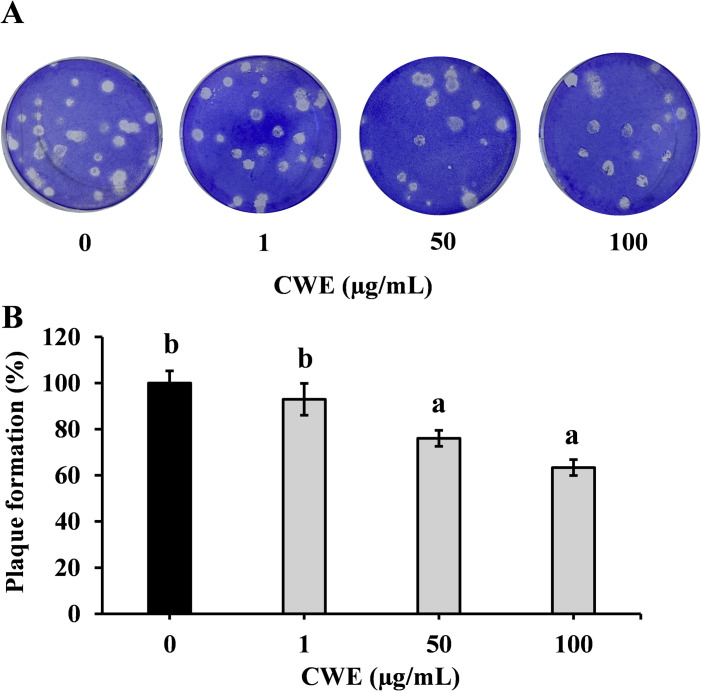
Effects of CWE on viral replication. The effect of CWE on IAV replication was determined by plaque formation assay. MDCK cells were pre-treated with CWE (1, 50, and 100 µg/mL) for 1 h before infecting the cells with IAV, and the infection time was also 1 h. Subsequently after infection, the cells were washed and overlaid with agar and incubated at 37^o^C for 72 h. (A) Representative images of plaque formations on pre-treated MDCK cells after infection and (B) plaque formation expressed as percentage compared with the untreated control (0 μg/mL). Data are presented as relative mean± SD of three independent experiments (n = 3) and compared with CWE-untreated control (0 μg/mL). Different letters denote a significant difference at *p* < 0.05.

As shown in Figs 7A and 7B, cells treated with 1 and 50 μg/mL CWE showed a 7% and 24% reduction in plaque formation, respectively, compared to the untreated control (0 μg/mL). Cells treated with 100 μg/mL CWE showed the greatest reduction in plaque formation (37%) compared to the untreated control (0 μg/mL).

### Contribution of cuminaldehyde to the inhibitory activity of CWE against IAV infection

**Fig 8:**
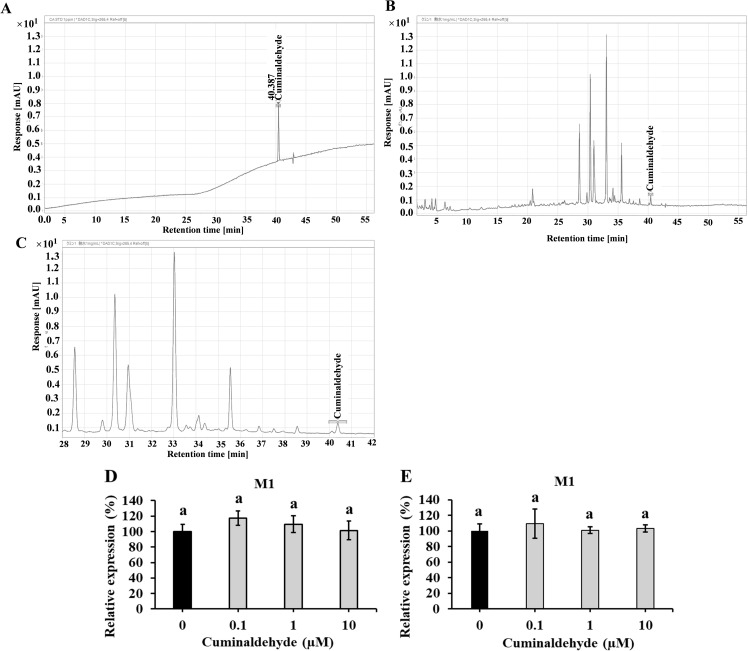
Effects of cuminaldehyde as a CWE component. (A) Chromatogram of CWE (1 mg/mL). (B) Enlarged chromatogram of CWE at 28–42 min (1 mg/mL). (C) Chromatogram of cuminaldehyde standard (1 ppm). Effects of cuminaldehyde at concentrations of 0, 0.1, 1, and 100 µM dissolved in 50% ethanol (D) or water (E) on IAV infection. Viral M1 protein expression in cells that received pre-infection treatment with cuminaldehyde standard. Data are presented as relative mean± SD against cuminaldehyde-untreated control (0 µM) (n = 3).

HPLC analysis of CWE was used to clarify the contribution of cuminaldehyde in the antiviral activity of CWE. As shown in the chromatograms, no clear peak (limit of detection is 1 ppm) of cuminaldehyde was identified at the retention time (Figs 8A and 8B) that was consistent with the peak of the reference cuminaldehyde (Fig 8C). On the other hand, other peaks were observed, presumably flavonoids and phenolic acids.

In addition, no inhibitory effect was observed in the pre-treatment test for IAV infection with cuminaldehyde dissolved in 50% ethanol (Fig 8D) or water (Fig 8E) at concentrations of 0.1, 1, and 10 μM.

## Discussion

Since ancient times, various types of spices have been utilised worldwide for food preservation, flavour enhancement, treatment of various disorders, and bolstering human immunity against life-threatening diseases. This is primarily because of the low cost of spices [19], their ability to increase patient tolerance, and their minimal residual effects. Furthermore, traditional plant and spice extracts have been used to treat viral infections [20]. Scientists have recently identified the effects of numerous bioactive components on viral infections [21] and have endeavoured to investigate the mechanisms of action of different bioactive components derived from various natural sources. In this study, we investigated whether CWE inhibits IAV infection.

Initially, we examined the effect of CWE treatment pre- and post-IAV infection on the inhibition of IAV replication. The results demonstrated a dose-dependent reduction in M1 levels, indicating that pre-infection treatment of cells with high concentrations of CWE reduced post-infection M1 levels. We also found that the concentrations of CWE used in this experiment showed no cytotoxic effects. M1 plays a crucial role in the process of budding, which involves the initiation, development, and release of viral buds with the assistance of host components [22]. Additionally, the M1 protein is located beneath the lipid layer and plays a role in the formation of a shell that connects the viral envelope with the nucleocapsid, forming the structure of the virion [23,24]. Inhibition of M1 has also been suggested to lead to failure of viral production [25], hinder the formation of new viral particles, and interfere with the movement of viral genetic material back into the cytosol, resulting in decreased viral infectivity and proliferation. GabAllah *et al.* performed an *in vitro* investigation using MDCK cells pre-treated with CWE and observed 44% suppression of the H5N1 virus [26]. Our results indicate that pretreatment of cells with CWE inhibits subsequent viral infection without adversely affecting the cells. We further showed that post-infection treatment of cells with CWE had no effect on the suppression of M1 proteins. This result suggests that CWE acts at an early stage of viral infection. After that, CWE pre-treated MDCK cells were used in a plaque formation test to verify the anti-influenza efficacy of CWE. The findings of the assay showed that CWE inhibited plaque development in a dose-dependent manner, which ultimately means that CWE has effects on influenza replication. Furthermore, compared with the untreated control (0 µg/mL), a 37% reduction of plaque formation was observed in cells treated with 100 µg/mL CWE.

Based on these results, we evaluated the inhibitory effect of CWE on the first step of viral infection, the adsorption process of the virus onto cells, via its erythrocyte agglutination inhibitory activity. CWE at concentrations ≥ 12.5 µg/mL inhibited haemagglutination. The first step in the viral infection cycle is the binding of viral HA to sialic acid receptors on the host cell surface. Through the interaction of the viral surface antigen HA and sialic acid receptors, influenza virus can enter the host cell [27]. Influenza virus infecting humans have binding sites for attachment with 2,6-sialic acids [28,29]. The haemagglutination-inhibiting activity of CWE suggests that it inhibits the attachment of the virus to the host, either by binding the antiviral component of CWE to the HA receptor binding site or by blocking the sialic acid-containing receptors on the host cell membrane. The inhibition of adsorption shown by the inhibition of red blood cell agglutination means that the interaction between the hemagglutinin on the virus and the sialic acid on the target cell was prevented by CWE. Since the agglutination of red blood cells does not occur with treatment with high concentrations of CWE alone, it is thought that CWE does not bind multivalently to sialic acid and competitively inhibit IAV adsorption.

After the attachment of the HA protein of the influenza virus with sialic acid, the virus enters the cell through the endocytic pathway and releases genomic components in the cytoplasm [30–32]. Thus, we next evaluated the effect of CWE on the entry process of the influenza virus. Like many other viruses, influenza viruses can enter cells via clathrin-mediated endocytosis [33], the major internalisation pathway for viruses [34–36]. To confirm that CWE had an anti-influenza effect, we performed an endocytosis inhibition assay using transferrin, a specific marker for clathrin-mediated endocytosis. Cells pre-treated with CWE showed reduced transferrin internalization. The endocytic pathway can be a good target for preventing viral entry into host cells, and several bioactive components help limit viral infection in the early stages of the viral life cycle [37] and reduce their infectivity [38]. The regulation of endocytic uptake of various viruses involves a variety of strategies, including membrane fluidity modulators that disrupt cell-cell fusion [39], intracytoplasmic entrapment of influenza virus on plasma membrane [40], and interference with endosome acidification [41]. All of these strategies restrict the entry of influenza virus [40–42]. Our results suggest that CWE can limit the clathrin-dependent influenza virus entry process into host cells. In addition, the method used to evaluate endocytosis in this study may be related to both the action of inhibiting the process of endosome formation and the action of acidifying endosomes. There are few natural materials that have been clearly reported to inhibit endocytosis, but ephedra herb, which is an extract of the rhizome of a plant in the Ephedraceae family and known as the component of Maoto in Chinese herbal medicine, has been reported to inhibit the infection of influenza virus PR8 strain by inhibiting the acidification of endosomes [43]. The mechanism of action is thought to be due to the inhibition of V-ATPase by tannins. However, tannins in CWE have not been confirmed, so we believe that further research is needed to clarify the specific mechanism of action.

In addition to the mechanisms described thus far, another possible mechanism by which CWE protects against infection may be by enhancing the resistance of target cells to viral infection. We determined whether CWE could stimulate the production of type I IFN, which is responsible for typical antiviral activity; type I IFN plays a crucial role in antiviral responses, limiting viral infection and protecting the host [44–47]. ISG15, Mx1, OAS1, and PKR are representative IFN-stimulated proteins that are activated by secreted IFNs and have broad antiviral properties [48]. The adhesion and invasion of the virus into the host cell are effects that are exerted before type I IFN is induced in the target cell by viral infection, and are thought to contribute to the suppression of CWE independent of IFN.

The active component of CWE that inhibits IAV infection has not yet been identified. Most of the research on the components of cumin seeds has been conducted on essential oil components, and research on the characterization of components obtained by hot water extraction is limited. Analyses of cumin fruits from multiple regions have reported that cuminaldehyde and oxygenated monoterpenes, such as 1,4-*p*-menthadiene-7-al, are predominant [49]. Cuminaldehyde is a major bioactive and characteristic compound of cumin, and has been suggested to possess inhibitory effects on several bacteria and viruses [50,51]. Therefore, we investigated the cuminaldehyde content in CWE and the anti-IAV activity of cuminaldehyde samples in the concentration range containing an authentic preparation of cuminaldehyde. Cuminaldehyde was not detected in CWE. Additionally, cuminaldehyde at concentrations 0.1–10 μM did not show antiviral activity. Therefore, the contribution of cuminaldehyde to the antiviral activity of CWE was negligible, suggesting that the antiviral activity was most likely due to other components. As shown in Fig 8, we also detected several unidentified peaks that are thought to be flavonoids and phenolic acids in CWE. Based on the results of previous research on hot water extracts of cumin fruits [52], substances such as luteolin and apigenin (flavonoids) and caffeic acid, ferulic acid, and p-coumaric acid (phenolic acids) were detected. In studies examining the effects of these compounds derived from other materials, it has been reported that luteolin, apigenin and ferulic acid show anti-IAV activity through various mechanisms, such as by reducing neuraminidase activity, whereas caffeic acid also inhibits IAV replication [53–57]. However, there are no reports of the antiviral activity of cumin water extracts, so it is necessary to carefully consider whether these components are present in CWE at the concentration levels at which they can exhibit inhibitory activity against IAV.

## Conclusion

**Fig 9:**
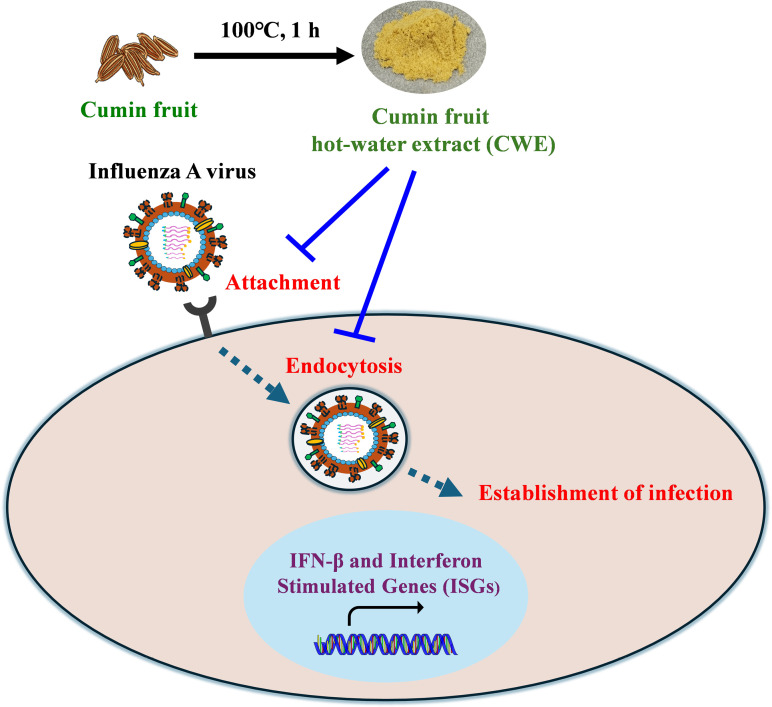
A schematic illustration of the mechanism by which CWE inhibits influenza A virus infection.

CWE exhibits promising antiviral activity against the influenza virus (H1N1) in MDCK cells, as demonstrated by the findings of the current study. However, it does not have cytotoxic effects against the host cells. Moreover, our findings indicate that CWE inhibits the attachment of viruses to host cells and their absorption by cells, hence preventing the spread of infection spread of infection (Fig 9). It is possible that cumin could be utilized as an antiviral agent to combat the infection caused by Influenza A virus; however, it’s *in vivo* effectiveness needs to be confirmed.
